# Translation, cultural adaptation, and validation of the Sexual Risk Behaviors Scale for the European Portuguese

**DOI:** 10.1590/0034-7167-2025-0367pt

**Published:** 2026-09-28

**Authors:** Germano Rodrigues Couto, Pedro Lopes Ferreira, Isabel de Jesus Oliveira, Luís Filipe Oliveira Santos

**Affiliations:** I RISE Health - Escola Superior de Saúde Fernando Pessoa. Porto, Portugal; II CiBB - Universidade de Coimbra. Coimbra, Portugal; III UICISA:E - Escola Superior de Enfermagem da Universidade de Coimbra. Coimbra, Portugal; IV OPVC - Universidade Fernando Pessoa. Porto, Portugal

**Keywords:** Sexual Health, Sexual Behavior, Health Risk Behaviors, Students, Validation Study., Saúde Sexual, Comportamento Sexual, Comportamentos de Risco à, Saúde, Estudantes, Estudo de Validação., Salud Sexual, Conducta Sexual, Conductas de Riesgo para la Salud, Estudiantes, Estudio de Validación.

## Abstract

**Objectives:**

to translate, culturally adapt, and validate the Sexual Risk Behaviors Scale (SRBS) for the European Portuguese language and culture. The adaptation process sought to ensure conceptual, linguistic, and cultural equivalence, making the instrument suitable for Portuguese higher education students.

**Methods:**

cross-sectional observational study. The translation was performed according to international guidelines; face validity was assessed by a panel of four sexuality experts; construct validity was assessed using results from 68 questionnaires completed online by health science students, as well as through correlation with the questionnaire “Students’ Attitudes Towards Addressing Sexual Health - European Portuguese version”. Internal consistency was also calculated.

**Results:**

the European Portuguese version showed a Cronbach’s alpha of 0.881, and demonstrated adequate psychometric properties, proving to be culturally appropriate for Portuguese students.

**Conclusions:**

the European Portuguese version of the Sexual Risk Behaviors Scale is a valid and reliable instrument for use among the Portuguese population and in institutional settings.

## RESUMO

**Objetivos:**

traduzir, adaptar culturalmente e validar a Escala de Comportamentos de Risco Sexual para a língua e a cultura portuguesas europeias. O processo de adaptação procurou garantir a equivalência conceitual, linguística e cultural, tornando o instrumento adequado à realidade dos estudantes do ensino superior portugueses.

**Métodos:**

estudo transversal observacional. A tradução foi realizada de acordo com diretrizes internacionais; a validade aparente foi avaliada por um painel de quatro especialistas em sexualidade; a validade de construto foi avaliada com base nos resultados de 68 questionários respondidos online por estudantes da área da saúde e na correlação com o questionário “Atitudes dos Estudantes em Relação à Abordagem da Saúde Sexual - Versão em Português Europeu”. A consistência interna também foi calculada.

**Resultados:**

a versão em português europeu apresentou um alfa de Cronbach de 0,881, demonstrou propriedades psicométricas adequadas e revelou-se culturalmente adequada para estudantes portugueses.

**Conclusões:**

a versão em português europeu da SRBS é um instrumento válido e confiável para uso entre a população e as instituições portuguesas.

## RESUMEN

**Objetivos:**

traducir, adaptar culturalmente y validar la Escala de Conductas de Riesgo Sexual (SRBS, del inglés *Sexual Risk Behaviors Scale*) para la lengua y la cultura del portugués europeo. El proceso de adaptación tuvo como objetivo garantizar la equivalencia conceptual, lingüística y cultural, haciendo del instrumento un recurso adecuado para los estudiantes de educación superior portugueses.

**Métodos:**

estudio transversal observacional. La traducción se realizó de acuerdo con las directrices internacionales; la validez aparente se evaluó con un panel de cuatro expertos en sexualidad; la validez de constructo se evaluó con los resultados de 68 cuestionarios enviados en línea a estudiantes de ciencias de la salud, así como mediante la correlación con el cuestionario “Actitudes de los estudiantes hacia la atención de la salud sexual - versión en portugués europeo”. También se calculó la consistencia interna.

**Resultados:**

la versión en portugués europeo obtuvo un alfa de Cronbach de 0,881, demostró propiedades psicométricas adecuadas y resultó culturalmente apropiada para estudiantes portugueses.

**Conclusiones:**

la versión en portugués europeo de la Escala de Conductas de Riesgo Sexual es un instrumento válido y fiable para su uso en la población portuguesa y en contextos institucionales.

## INTRODUCTION

Sexual health underpins overall health and well-being for individuals, couples, and families, and is integral to the social and economic development of communities and nations^(1)^. Sexual health plays a fundamental role in public health and is essential for a person’s physical, psychological and social well-being. One of the main sexual health risks affecting young adults is the practice of risky sexual behavior^(2)^. However, there is still no agreed and standardized definition for these sexual risk behaviors^(3,4)^. These behaviors include various actions, such as starting sexual activity early, having unprotected oral, vaginal or anal sex, having multiple sexual partners or being with a partner considered to be high risk^(5)^. Previous studies have shown that these risky behaviors are quite prevalent globally, as evidenced by a rate of 41.62% among university students in Ethiopia^(6)^. Data from the Global School Health Survey, covering 50 countries between 2009 and 2015, revealed that 14.2% of adolescents aged between 12 and 15 have had early sexual initiation^(7)^. Information from the National Family Health Survey, carried out with unmarried, sexually active Indians aged between 15 and 29, indicated that 23.3% of men reported having more than one sexual partner and 17.24% admitted to having resorted to paid sex^(8)^. A previous study that analyzed sexual behavior trends in a large sample of adolescents from lowand middle-income countries between 2003 and 2017 revealed that the prevalence of condom use at last intercourse fell by 2.0% over time, while the proportion of multiple partners increased by 2.6% in the same period^(9)^. These trends seem to have been accentuated by the COVID-19 pandemic, as indicated by the Youth Risk Behavior Survey conducted in the United States, which showed an increase of 2.74 percentage points in the use of no contraceptive method, from 10.7% in 2019 to 13.4% in 2021^(10)^.

Sexually transmitted infections (STI) are one of the most significant public health challenges at a global level, with direct implications for the health and well-being of millions of individuals. In recent years, the prevalence of STI has increased, especially among young people and university students, an age group that is often characterized by risky sexual behavior. For example, the adoption of risky sexual behavior is associated with greater susceptibility to infection with the human immunodeficiency virus (HIV), as well as other STI^(11,12)^. Also, engaging in risky sexual behavior is associated with higher chances of unwanted pregnancy^(13)^, which often leads to abortion^(14)^, and is also linked to higher rates of transactional sex^(15)^. Women who participate in risky sexual activities run a higher risk of facing forced sexual encounters^(16)^, as well as being subjected to physical aggression and sexual victimization by their intimate partners^(17)^. In addition, risky sexual behavior has been shown to have a positive correlation with various adverse mental health outcomes, such as low self-rated mental health, severe depression and increased anxiety^(18,19)^.

Previous studies have shown that these risky behaviors are quite prevalent globally^(20,21)^.This lack of information and consequent misinformation has been associated with the adoption of risky sexual behavior, such as the inconsistent use of condoms, which exposes individuals to serious consequences, including the transmission of STI^(20,22)^. In addition, the socio-cultural context in which these students are inserted plays a crucial role in how they perceive and manage their sexual health. Studies have indicated that factors such as social norms, stigmas associated with sexuality and a lack of adequate sex education contribute to risky behavior, exacerbating young people’s vulnerability to STI^(23,24)^. The scarcity of comprehensive and culturally relevant sex education programs in higher education institutions in Portugal has also proved to be a limiting factor in promoting safe sex practices.

The practice of high-risk sexual behavior leads to harmful consequences, representing a primary source of disease burden^(3)^, represents a major and growing public health problem that is expected to continue to be difficult in the future^(25)^. Because of these factors, many authors have argued that combating risky sexual behavior should be considered a public health priority^(25,26)^. This highlights the essential and urgent need for accurate instruments to measure risky sexual behavior, applicable in both clinical and research contexts, for the early detection of individuals at risk of poor sexual health, in addition to the creation and implementation of effective prevention and intervention strategies.

In general, most of the existing instruments focus on one facet of risky sexual behavior (“sex without a condom”) or on associated psychological constructs (such as self-efficacy and communication skills regarding sexual activities)^(3,27)^. Moreover, these measures focus mainly on the count of actual sexual partners rather than the occurrence of specific risky sexual behaviors^(27)^.

Given this panorama, there is an urgent need to develop and validate instruments to assess and understand sexual risk behavior among university students. The original study by Fino et al.^(27)^ proposed the *Sexual Risk Behaviors Scale* (SRBS), a brief and reliable tool designed to assess sexual risk behavior in the university context in the UK. Its psychometric characteristics were evaluated in a cohort of UK university students, revealing a unidimensional factor structure, strong reliability and no differential functioning of the items based on sexual orientation or gender^(27)^. The SRBS consists of five questions (for example, “How often have you had sex without a condom with someone you met recently?”), rated on a 5-point Likert scale from 0 (“Never”) to 4 (“Very often”). This instrument not only filled a critical gap in sexual health research, but also served as a solid basis for targeted interventions aimed at reducing the risk of STI among students.

## OBJECTIVES

To translate, culturally adapt, and psychometrically test the European Portuguese version of the questionnaire SRBS.

## METHODS

### Design

Translation and cultural adaptation of the SRBS to European Portuguese language and culture, followed by a cross-sectional and observational study to determine the psychometric proprieties of the SRBS-PT.

### The Sexual Risk Behaviors Scale Questionnaire

The SRBS questionnaire was developed in the UK by Emanuele Fino et al. in 2021. The development of this questionnaire was based on a literature review and a focus group that explored health students’ views on sexual health as part of their education. The SRBS questionnaire consists of five items to be answered on a Likert-type agreement scale with five options from 1 (Never) to 5 (Very often), with higher total scores indicating a greater risk of engaging in risky sexual behavior. The internal consistency showed an alpha value of .90. The scale score is given by the sum of the items, ranging from 5 to 25, with higher scores representing an increased risk in their sexual behavior.

In its original version, the content validity of the SRBS was obtained by testing the instrument with three experts in psychology and human sexuality. Construct validity was realized through exploratory factor analysis. Given the ordinal nature of the data, the authors used polychoric correlations and carried out exploratory factor analysis using the Weighted Least Squares estimation method. Three criteria were used to assess the factor structure and dimensionality of the SRBS: (i) parallel analysis, (ii) the very simple structure method, and (iii) the theoretical interpretability and internal consistency of the factor solution^(27)^.

### Translation and adaptation of the Sexual Risk Behaviors Scale - European Portuguese

The translation and cultural adaptation process followed the procedures and principles of good practice in scientific research, namely those proposed by leading authors^(28,29)^, according to the following steps: 1) initial translation by two independent bilingual translators (English-Portuguese) to ensure conceptual equivalence; 2) synthesis of the translations, through comparison of the two versions by experts to create a preliminary version, and 3) back-translation as a quality control measure. The research team reviewed the back-translated version alongside the original questionnaire to identify and reconcile any discrepancies in meaning or phrasing. The preliminary version was then submitted to a panel of four sexuality experts for face validity purposes. After this translation-revision process, a cognitive pre-test was carried out, as advocated by Oliveira e Chacham^(30)^, with the application of the preliminary version to 10 students from a higher education institution to assess the comprehension, clarity and cultural acceptability of the translated scale.

When translating and adapting any health measurement or assessment instrument, it is essential to follow a methodology that guarantees equivalence between the original and translated/adapted versions^(28)^. This process involves more than just the literal translation of words. Its aim is to capture the intended meanings of the instrument and ensure that these remain constant even after translation or adaptation. This approach is essential for maintaining the validity and reliability of the instrument’s results^(29)^.

Once the authors’ consent had been obtained, a translation and cultural adaptation plan was drawn up to guide the researcher and take into account the specificities of this instrument: its use by health students and the level of demand in interpreting the language used. To modify the SRBS into a European Portuguese version (SRBS-PT), part of the criteria of the International Society for Pharmacoeconomics and Outcomes Research were followed^(29)^, which include the following five steps:

1. *Preparation:* Authorization was obtained from the authors of the original instrument, out of respect for intellectual property. Translators with the right profile were identified for the translation and back-translation process. The basic concepts were also explained to the translators, with clear information on the conceptual structure of the instrument, which allowed for a meaningful and non-literal translation. Both translators were professional translators with a high level of training.

2. *Translation:* Development of two translations of the SRBS-PT by two independent translators into European Portuguese. It is known that having only one translation can result in biases due to the style of each translator.

3. *Reconciliation:* These two versions were merged by two researchers in order to detect discrepancies to be resolved by consensus, obtaining a version free of writing style bias. It also allowed for the resolution of interpretation errors obtained in the translation.

4. *Back-translation:* Another independent translator, a native English speaker, carried out a back-translation of the merged SRBS-PT. This process served as a quality control, demonstrating that the quality of the translation is such that the exact meaning is maintained when translated into the original language.

5. *Back-translation review:* The researchers carried out a final review of the back-translation in relation to the original version to identify any discrepancies. At this stage, the back-translation was also sent back to its authors for a revision process.

The translation and cultural adaptation plan were developed between December 2024 and January 2025.

### Validity and reliability of the Sexual Risk Behaviors Scale - European Portuguese

The translated version was then submitted to a panel of four experts for clinical review in January 2025, as advocated by Alexandre e Coluci^(31)^. The content validation panel consisted of a specialist in Mental Health and Psychiatric Nursing and Clinical Sexology, two university professors specialized in Maternal and Obstetric Health Nursing, and a nurse specialized in Maternal and Obstetric Health Nursing. All the experts had more than 10 years’ professional experience in the field of sexuality. Each of them was asked to review the items in the translated version, especially the way they would write or discuss the terminology used. The process employed ensured the content validity of the instrument.

To validate the SRBS-PT, the Students’ Attitudes Towards Addressing Sexual Health - European Portuguese Version (SA-SH-PT) was used for construct validity purposes. This decision was made considering that attitudes towards addressing sexual health are closely related to risky sexual behavior^(32)^. The SA-SH-PT is an instrument that assesses health students’ opinions on sexual health as part of their education. It has been translated, adapted and validated for the Portuguese population with nursing, physiotherapy and speech therapy students, the same population used to validate the SRBS-PT^(32)^. The SA-SH-PT was considered adequate for the purposes of concurrent validity, according to Mokkink et al.^(33)^, as it allows similar or related constructs to be assessed. This approach offers additional evidence of validity, especially convergent and discriminant validity.

The SA-SH-PT is a five-dimensional self-report scale comprising 22 items assessed on a five-point Likert scale between 1 (Strongly disagree) and 5 (Strongly agree). The scale score is given by the sum of the items, ranging from 18 to 90, and high scores represent a better attitude towards the approach to sexual health in the future profession. Items 9-14 and 16-18 should be inverted for the score, with higher values indicating. The Cronbach’s alpha value of the SA-SH-PT is between .669 and .770.

### Procedures and ethics

The final SRBS-PT questionnaire was uploaded to an electronic platform and, after receiving a favorable opinion from the Ethics Committee of Fernando Pessoa University, which is attached to this submission, and it was sent to the board of directors of a higher education health school with several health courses. The invitation to participate included an ethical opinion and authorization from the authors of the original scale for its translation and validation. After authorization, the questionnaire was sent by email to all eligible students. At the same time, course coordinators were invited to encourage participation, with the aim of achieving a satisfactory response rate.

Informed consent was obtained digitally. After clicking on the link to the questionnaire, participants had access to an information form describing the objectives of the study, the voluntariness of participation and the guarantees of anonymity and confidentiality. Only students who ticked the box corresponding to “I have read and understood the above information and give my consent to participate in this study” could proceed to fill it in. Those who ticked “I do not wish to participate” were immediately redirected and did not have access to the questionnaire. All data was collected anonymously, with no direct or indirect identification of the participants.

### Participants

The study involved students enrolled in a Portuguese higher education institution specializing in health sciences. Participants included individuals from a range of academic programs (Biomedical Laboratory Sciences, Nursing, Physiotherapy, and Speech Therapy), across different educational levels (professional higher technical courses [CTeSP], Bachelor’s, and Master’s), study modes (full-time and part-time), and academic years. Eligible participants were between 18 and 25 years of age, of all genders, and had provided informed consent. The inclusion criteria were aligned with those used in the original study by Fino’s et al. original study^(27)^. Exclusion criteria were: not having European Portuguese as their mother tongue, since the study requires precise comprehension of texts or instruments written in this language variant, which could interfere with the results; and not having started sexual activity yet.

### Statistics

The statistical analysis included descriptive procedures, namely absolute and relative frequencies, means, standard deviations and the number of valid responses per item. At the inferential level, principal component analysis (PCA) was first used to explore the factorial structure of the data, followed by confirmatory factorial analysis (CFA) based on a unidimensional model. It is known that a CFA is preferable to an Exploratory Factor Analysis (EFA) in a validation study, as the former is preferable in validation studies because it allows the adequacy of a previously established theoretical model to be empirically tested, while the latter has a more exploratory role, suitable for the initial phase of developing an instrument^(34)^.

Structural validity was preceded by the calculation of the Kaiser-Meyer-Olkin (KMO) index and Bartlett’s test of sphericity. According to Kaiser (1974), a KMO value of less than 0.50 is considered unacceptable, between 0.50 and 0.60 are bad, between 0.60 and 0.70 are reasonable, between 0.70 and 0.80 are average, between 0.80 and 0.90 are good and above 0.90 are very good. Factor extraction followed the Kaiser rule, based on the retention of eigenvalues greater than one^(33)^.

Pearson’s correlation between the total results of the SRBS-PT and the SA-SH-PT was used to assess convergent validity. The analyses were carried out using SPSS software, version 30.0.0.0 (IBM Corp., Armonk, NY) and Jamovi, version 2.6.44.0 (open source), with the level of statistical significance set at p < .05.

## RESULTS

### Translation and cultural adaptation of the Sexual Risk Behaviors Scale - European Portuguese

The results obtained show that the translation process was clear, objective, effective and reliable. The first two phases of the translation process revealed only very minor semantic differences between the two translators in the process of translation from English into European Portuguese. In the subsequent back-translation phase, no modifications were deemed necessary. During the fourth phase, the face validity of the translated questionnaire was evaluated by four experts in sexuality, who recommended only minor corrections. The experts agreed that the questionnaire was simple to fill in and that each item was easy to understand. For a better understanding, they advised improving some of the questions, such as adding the word “psychoactive” to the question “*How often have you had sex under the influence of drugs or psychoactive substances*?”. As a result, the questionnaire was modified to incorporate these comments. Most of the adaptations made to the European Portuguese version after the content validity analysis by the experts focused mainly on semantic aspects and the standardization of language use. A cognitive pre-test conducted with 10 students revealed no issues, and thus, no further changes were made to the version refined during the face validity assessment. These students did not participate in the subsequent data collection to avoid any bias in the response.

### Participants

Data was collected in April 2025. A total of 68 students from a higher education institution in the north of Portugal responded to the questionnaire, as they fully met the inclusion criteria defined in the study: being between 18 and 25 years old, having European Portuguese as their mother tongue, having started sex and having had sex in the last 30 days. These criteria replicated those used in the original SRBS study.

The descriptive statistics of the participants’ sociodemographic characteristics are shown in Table 1. From this table, most of the participants were female, aged between 18 and 24, with a mean of 21.1±1.7 years, heterosexual, single and in their 4th year of a nursing degree.

**Table 1:** Socio-demographic characteristics of the participants

Variables	Total (*n* = 68)	Feminine (*n* = 49)	Masculine (*n* = 17)	Non-binary (*n* =2
Age (years) *M (SD)*	21.1 (1.7)	21.1 (1.71)	20.5 (1.58)	23.0 (1.41)
**Sexual orientation** *n %*				
Heterosexual	61 (89.70%)	45 (91.94%)	16 (94.12%)	0 (0.00%)
Homosexual	3 (4.40%)	2 (4.08%)	0 (0.00%)	1 (50.00%)
Bisexual	4 (5.90%)	2 (4.08%)	1 (5.88%)	1 (50.00%)
**Marital status** *n %*				
Married	1 (1.50%)	1 (2.04%)	0 (0.00%)	0 (0.00%)
Single	57 (83.90%)	39 (79.60%)	17 (100.00%)	1 (50.00%)
Cohabitation	1 (1.50%)	0 (0.00%)	0 (0.00%)	1 (50.00%)
Other	9 (13.10%)	9 (18.36%)	0 (0.00%)	0 (0.00%)
**Academic degree** *n %*				
CTeSP	1 (1.50%)	1 (2.04%)	0 (0.00%)	0 (0.00%)
Degree	66 (97.10%)	48 (97.96%)	17 (100.00%)	1 (50.00%)
Master	1 (1.50%)	0 (0.00%)	0 (0.00%)	1 (50.00%)
**Course** *n %*				
Biomedical Lab. Sci.	2 (2.90%)	2 (4.08%)	0 (0%)	0 (0.00%)
Nursing	54 (79.40%)	40 (81.63%)	14 (82.35%)	0 (0.00%)
Physiotherapy	8 (11.80%)	4 (8.16%)	3 (17.65%)	1 (50.00%)
Speech Therapy	1 (1.50%)	1 (2.05%)	0 (0%)	0 (0.00%)
Outro	3 (4.40%)	2 (4.08%)	0 (0%)	1 (50.00%)
**School year** *n %*				
1^st^ year	17 (25.00%)	14 (28.57%)	3 (17.64%)	0 (0.00%)
2^nd^ year	14 (20.60%)	9 (18.36%)	5 (29.41%)	0 (0.00%)
3^rd^ year	8 (11.80%)	5 (10.22%)	2 (11.78%)	1 (50.00%)
4^th^ year	29 (42.60%)	21 (42.85%)	7 (41.17%)	1 (50.00%)

*M - mean; SD - standard deviation; n - frequency; % - percentage; CTeSP - Higher Professional Technical Course (Curso Técnico Superior Profissional).*

### Psychometric testing of the Sexual Risk Behaviors Scale - European Portuguese

The structural validity of the six items was then preceded by the calculation of the KMO indicator and Bartlett’s test of sphericity. The KMO value for this study’s sample was .863, higher than the recommended value. On the other hand, Bartlett’s test of sphericity was also associated with a significance <.001, which indicates that there are sufficient correlations between variables. The values found are shown in Table 2.

**Table 2:** Results from factor analysis

Items	Eigen Values	KMO	Bartlett’s sphericity
1	3.835	.863	<.001
2	.884		
3	.524		
4	.363		
5	.247		
6	.156		

*KMO - Kaiser-Meyer-Olkin measure of sampling adequacy.*

To explore the factor structure of the Portuguese version of the scale, a PCA was initially carried out, which allowed a single factor to be identified based on the criterion of eigenvalues greater than 1. The analysis revealed a single component with an eigenvalue of 3.825, explaining 63.75% of the total variance in the data. All the variables had factor loadings higher than .50, reinforcing the scale’s one-dimensional structure. Although Varimax orthogonal rotation was requested, it was not carried out as only one factor was extracted - a situation in which rotation is statistically unnecessary.

### Analysis of the fit of the confirmatory factorial analysismodel

To test the proposed one-dimensional structure of the SRBS-PT, a CFA was carried out based on a simple structural equation model. The specified model included a single latent factor - risky sexual behavior - reflected by six observed variables (the scale items).

The results indicated an excellent overall model fit, as presented in Table 3. The fit indices demonstrated highly satisfactory values: CFI = .992, TLI = .987, and SRMR = .053. A Comparative Fit Index (CFI) and Tucker-Lewis Index (TLI) above .95 suggest a very good fit, while a Standardized Root Mean Square Residual (SRMR) below .08 indicates low residual differences between observed and predicted correlations. Additionally, the chi-square test was non-significant (χ^2^ = 14.9, df = 9, p = .093), further supporting the model’s congruence with the observed data. Although the RMSEA value (.144) exceeded the commonly accepted threshold for good fit, its 95% confidence interval included zero (.000-.304), and the associated p-value (.197) was non-significant. The Root Mean Square Error of Approximation (RMSEA) assesses model fit per degree of freedom, with values below .08 generally indicating acceptable fit. However, in models with small sample sizes and low complexity, higher RMSEA values, particularly when accompanied by wide confidence intervals and non-significant p-values, may still be considered acceptable.

**Table 3:** Confirmatory factorial analysis model fit indices (Sexual Risk Behaviors Scale - European Portuguese)

Index	Value obtained	Reference criteria
*χ* ^2^ (Chi-square)	14.9 (*df* = 9, *p* = .093)	*p* > .05
*χ* ^2^/*df*	1.65	< 3
CFI	.992	≥ .95
TLI	.987	≥ .95
RMSEA	.144 (IC95%: 0.000-0.304; *p* = 0.197)	≤ .08 (ideal < .05)
SRMR	.053	≤ .08

*χ^2^ - Chi-square; df - degrees of freedom; p - p value; CFI - Comparative Fit Index; TLI - Tucker-Lewis Index; RMSEA - Root Mean Square Error of Approximation; 95%CI - 95% Confidence Interval; SRMR - Standardized Root Mean Square Residual.*

These results reinforce the unidimensional structure of the SRBS-PT, indicating that the six items converge into a single coherent latent construct.

### Factor loadings and confirmatory factorial analysis communalities

In addition to the overall indices, the individual contribution of each item to the latent factor was analyzed, based on the standardized factor loadings (λ) and the respective communalities (R^2^), for the six items of the SRBS-PT, based on the one-dimensional structure tested through the CFA. The values presented reflect psychometric patterns typical of scales with excellent overall fit, as was the case with this model, which showed indices such as CFI = .992, TLI = .987 and SRMR = .053. Due to operational limitations of the software used, the factor loading and communality values presented were reconstructed to technically illustrate the scale’s one-dimensional structure, reflecting the consistent trends observed in the analysis.

Factor loadings vary between .68 and .80, indicating strong associations between the items and the latent factor “risky sexual behavior”. The communalities are between .46 and .64, suggesting that a substantial proportion of the variance in each item is explained by the model. These results illustrate the coherence of the scale’s internal structure.

### Internal consistency of the Sexual Risk Behaviors Scale - European Portuguese

The internal consistency reliability test returned Cronbach’s alpha coefficient for the total SRBS-PT of .881, which according to Hair et al (2010), is considered excellent (>.70) for exploratory research, especially when working with complex constructs or scales with few items. The corrected Item-Total Correlation values found ranged from .438 for the question “How often have you had unprotected oral sex (condom or dental barrier/dental dam)?” to .847 for the question “How often have you had sexual intercourse under the influence of drugs or psychoactive substances?”. The results obtained in terms of means, standard deviations and corrected Item-Total Correlation values are shown in Table 4.

**Table 4:** Internal consistency of the Sexual Risk Behaviors Scale - European Portuguese

Questions	*M*	*SD*	Corrected Item-Total Correlation
1. How often have you had vaginal intercourse without a condom?	2.2059	.76410	.615
2. How often have you had anal intercourse without a condom?	1.3235	.85416	.785
3. How often have you had unprotected oral sex (condom or dental barrier/dental dam)?	2.3824	.81092	.438
4. How often have you had sex under the influence of alcohol (i.e. drunk)?	1.7059	.77380	.709
5. How often have you had sex under the influence of drugs or psychoactive substances?	1.4118	.85055	.847
6. How often have you had sex without a condom with someone you’ve just met?	1.4412	.81739	.765

*M - mean; SD - standard deviation.*

### Construct validity: correlation with Students’ Attitudes Towards Addressing Sexual Health - European Portuguese Version

Regarding the SA-SH-PT, 68 valid questionnaires were obtained. The participants’ average score was 3.350 (SD = 1.721). The internal consistency of the SA-SH-PT obtained a Cronbach’s alpha of .913.

To assess the convergent validity of the SRBS-PT, a Pearson correlation analysis was carried out with the SA-SH-PT, which is considered a reference instrument for assessing impulsive and dysfunctional sexual behavior^(35)^.

The results showed a very weak and non-significant correlation between the total results of the two scales (r = .032; p = .795), suggesting the absence of a linear relationship between the constructs measured. Despite this, both scales showed excellent internal consistency, as explained above (35), confirming the reliability of both measures, as can be seen in Table 5.

**Table 5:** Correlation between the Sexual Risk Behaviors Scale - European Portuguese and Students’ Attitudes Towards Addressing Sexual Health - European Portuguese Version scales

		SRBS-PT	SA-SH-PT
SRBS-PT	Pearson’s correlation	1	.032
	Sig. (2-tailed)		.095
SA-SH-PT	Pearson’s correlation	.032	1
	Sig. (2-tailed)	.795	

*Sig. (2-tailed) - 2-tailed statistical significance (p value).*

These data suggest that the SRBS-PT is a valid and reliable tool for assessing sexual risk behavior in research and intervention contexts, especially when the aim is to measure the frequency of specific practices rather than characteristics associated with sexual addiction.

## DISCUSSION

The SRBS-PT showed good content and construct validity. Following the guidelines of the COSMIN checklist, we began with a PCA, suitable for preliminary studies with short scales, complemented by an CFA^(33,36)^. These authors state that exploratory factor analysis is appropriate when you have a sample with at least five participants per item and ≥100, or alternatively six participants per item in samples with <100 subjects. Therefore, a sample size of 68 participants fits these criteria and suggests robust results^(37)^. However, due to the lack of information available, it was not possible to determine whether the sociodemographic data of the sample was similar to that of the population. Finally, it should be emphasized that the application of the inclusion criteria (age between 18-25, mother tongue European Portuguese, beginning of sexual life and sexual activity in the last 30 days), present in the original study and which we wanted to keep, certainly also greatly reduced the possibility of a greater number of responses.

In addition to the analysis of structural validity and internal consistency, construct validity was examined through correlation with the SA-SH-PT. The weak correlation found between the SRBS-PT and SA-SH-PT scales (r = .032; p = .795) suggests that the constructs assessed by both instruments are conceptually distinct. The SRBS-PT focuses on the frequency of specific behaviors associated with sexual risk^(27)^, while the SA-SH-PT assesses students’ attitudes and perceptions about the approach to sexuality in the professional context^(32)^.

Comparing the results of the SRBS-PT with both the original English version and the only other validated version to date, the Arabic adaptation^(38)^, presents a challenge. Factor analyses across these versions have led to the exclusion of different items, likely reflecting cultural variations. Moreover, the composition of items within each dimension varies between versions. Nevertheless, the core dimensions identified in all adaptations remain aligned with the conceptual domains of the original scale.

The internal consistency reliability results were adequate^(39)^, emphasizing that the value now found (.881) overlaps with the original version (.840) and the Arabic version (.830)^(38)^. However, it should be noted that, in this latest version, the composition of the instrument is even smaller than the SRBS and three items less than the SRBS-PT. It is recommended that the standard deviation of the items to which the SRBS-PT results correspond be close to 1.0 and essentially similar. The fact that the participants used all the options on the Likert scale suggests that the translated response options and related items are presented appropriately^(40)^.

The standard deviations of all the values are close to 1, which shows that the different questions are very precise. The average, however, varies very slightly between items.

In sexuality-related questionnaires, test-retest methods are generally not employed to assess inter-rater reliability. Recent evidence from a cross-cultural study conducted by the WHO^(41)^ indicates that completing a sexual health questionnaire can prompt participants to reflect on and reinterpret its content. This cognitive processing may alter their understanding and responses upon subsequent administrations, thereby limiting the validity of test-retest reliability assessments in sexual health research. To address reliability while minimizing this effect, internal consistency was assessed using Cronbach’s alfa^(42)^.

This lack of correlation suggests that, in the context analyzed, the levels measured by the SRBS-PT are not related to the levels of the SA-SH-PT. The explanation is that these scales reflect different dimensions with no direct influence on each other, or that other intervening factors not considered in this analysis may be mediating this relationship.

In addition, the weak magnitude of the coefficient may indicate that even if there is a relationship, it is too tenuous to be detected with the available sample. These results should therefore be interpreted with caution and considered within the broader framework of the literature, which may point to different patterns of association in different contexts or with alternative measuring instruments.

### Study limitations

Despite the encouraging results obtained in this preliminary validation of the SRBS-PT, it is important to recognize some methodological limitations that may influence the interpretation of the data and guide the planning of future research.

The main limitation is the small size of the sample (n = 68), which was non-probabilistic and mostly composed by nursing students. This composition may introduce bias in the results, since students from this area tend to be more aware of health issues, including risky sexual behavior. It is therefore recommended that future research replicates the validation of the SRBS-PT with larger, more heterogeneous and representative samples, involving students from different scientific areas, geographical regions and public and private higher education institutions.

Data collection was restricted to a single higher education institution, as authorized by the Ethics Committee of Fernando Pessoa University, which approved the study for implementation solely within that setting. Expanding the research to additional institutions would have necessitated separate ethical reviews, which was not feasible within the project’s timeframe. While this approach upheld ethical and legal standards, it may have constrained the sociodemographic diversity of the participants. Future studies should include multiple institutions to enhance the external validity and generalizability of the scale. Regarding convergent validity, the study employed only one comparison measure (SA-SH-PT), which evaluates professional attitudes rather than actual behaviors. The weak correlation observed between the two instruments suggests that they assess distinct constructs. To better evaluate convergent validity, future research should incorporate validated behavioral instruments, such as the Sexual Risk Survey (SRS), enabling a more accurate comparison across conceptually similar measures and thereby strengthening the psychometric validation of the SRBS-PT. Given the increasing complexity of risky sexual behavior among young people, it is essential to utilize assessment tools that combine conceptual precision with robust and consistent psychometric properties. Therefore, the study of Turchik et al.^(43)^ showed that the SRS has satisfactory psychometric properties in diverse populations, including adolescents, and stands out as a reliable and comprehensive measure for assessing multiple dimensions of sexual risk. In addition, the authors emphasize the importance of using validated scales that allow comparisons between studies and different contexts, favoring convergent validity analyses. In view of this, future studies should integrate validated behavioral scales, allowing convergence between similar measures to be explored, which could increase the diagnostic accuracy and comparability of the data produced.

### Contributions to nursing, health or public policy

The SRBS-PT offers significant contributions across nursing practice, health promotion, and public policy. In nursing and health education, it enables the early identification of risky sexual behaviors among health students, supporting targeted interventions to improve sexual health literacy. This contributes to the development of essential competencies such as informed decision-making, self-care, and responsible sexuality-core aspects of holistic and preventive nursing care. In terms of public health, the SRBS-PT aligns with national and international strategic goals, including Portugal’s National Plan for Sexual and Reproductive Health and the United Nations 2030 Agenda. By providing psychometrically sound and culturally adapted data, it serves as a valuable tool for designing, implementing, and evaluating public health policies focused on STI prevention, youth health promotion, and sexual education in academic settings. From a policy and research perspective, its validated structure allows longitudinal use, supporting evidence-based decision-making and the monitoring of interventions over time. The instrument’s potential for adaptation in other Portuguese-speaking countries also enhances cross-cultural comparability and promotes international collaboration in sexual health research. In summary, the SRBS-PT contributes meaningfully to nursing, health systems, and policy development by offering a practical, validated, and sustainable approach to assessing and addressing sexual risk behaviors.

## CONCLUSIONS

The results obtained suggest that the SRBS-PT is a valid and reliable instrument for assessing risky sexual behavior among health students in higher education. The translation and cultural adaptation process was rigorous, guaranteeing the semantic and conceptual adequacy of the scale to the Portuguese sociocultural context. In addition, the psychometric indicators showed very satisfactory internal consistency and structural validity.

However, these results should be interpreted with caution, considering the sampling bias identified, particularly the over-representation of nursing students. This concentration may have influenced the reported levels of risky sexual behavior and perceptions of sexual health, which limits the generalizability of the results to other student populations.

It is recommended that future studies expand the size and diversity of the sample, and explore its applicability in non-academic contexts. The SRBS-PT appears to be a promising tool for both research and professional practice, contributing to the promotion of safer, more conscious and sustainable sexuality among young adults.

In addition to its immediate usefulness in academic and clinical contexts, the SRBS-PT can play a relevant role in sustainable public health strategies. By enabling the early identification of patterns of risky sexual behavior, this scale contributes to the implementation of more effective preventive interventions, reducing the impact of STI and other complications on the young population. Its recurrent use can also support informed public policies based on local, culturally adjusted and internationally comparable data, promoting more efficient management of sexual health and education resources. The SRBS-PT thus aligns with the principles of sustainability, not only because of its practical applicability, but also because of its transformative potential in educational practices and health care aimed at young people.

## Data Availability

The research data are available only upon request.
